# Pilot study of the effectiveness of a telehealth group for improving peer relationships for adolescents with neurofibromatosis type 1

**DOI:** 10.1186/s13023-024-03093-1

**Published:** 2024-03-12

**Authors:** Danielle M. Glad, Sara K. Pardej, Ellen Olszewski, Bonita P. Klein-Tasman

**Affiliations:** Department of Psychology, University of Wisconsin– Milwaukee, 2441 E Hartford Ave, Wisconsin 53211 Milwaukee, USA

**Keywords:** Neurofibromatosis type 1, NF1, Telehealth, Virtual, PEERS®, Social

## Abstract

**Background:**

Interventions for social difficulties have not been investigated in the neurofibromatosis type 1 (NF1) population despite observations of elevated rates of social difficulties. In this pilot study, the effectiveness of a 14-week telehealth PEERS® intervention with nineteen adolescents with NF1 (*M*_*age*_=13.79 years, *SD* = 1.32) with social skills difficulties was examined. Measures of social outcomes were completed at three timepoints (before, immediately after, and at 14-week follow-up).

**Results:**

Caregiver-reported social-emotional skills, social impairment, caregiver-reported number of adolescent get-togethers, and teen social knowledge showed significant improvement following the intervention.

**Conclusions:**

The PEERS® intervention is promising to support the social and friendship skills of adolescents with NF1 who have social difficulties.

## Background

Social functioning difficulties are one area of vulnerability for individuals with neurofibromatosis type 1 (NF1). NF1 is a neurogenetic condition resulting from a pathogenic variant of the NF1 gene which encodes for the tumor suppressor protein, neurofibromin. NF1 is associated with characteristic physical manifestations including cutaneous neurofibromas and café-au-lait spots, and vulnerability to plexiform neurofibromas, skeletal abnormalities, and optic gliomas [1–3]. Specific challenges have been described for social skills and social outcomes [4–7], social competence [4, 8, 9], and social problems [2–5, 7, 8, 10, 11]. One study using a peer nomination approach found that children with NF1 are more sensitive, more socially isolated, show less leadership behavior, are chosen as a best friend less often, have fewer reciprocated friendships, and are less liked in comparison to classroom peers [12]. In the only published psychosocial intervention research with individuals with NF1, a virtual mind-body intervention that incorporates relaxation strategies and coping skills showed improvements in social quality of life within NF1 [13–15]. Even though social difficulties are observed for many children and adolescents with NF1 [4–7], continue into adulthood [16] and may be more prominent with age [17], interventions specifically designed to improve social and friendship skills, have not been explored. Given the benefits of having social relationships and the consequences of social difficulties, it is important to understand how to intervene during adolescence to promote better social outcomes.

Individuals with NF1 show elevated rates of attention problems and autism spectrum disorders (ASD) in comparison to the general population, both of which relate to social functioning difficulties. Approximately 30–50% of children with NF1 meet criteria for attention-deficit/hyperactivity disorder (ADHD) [18–21]. As is the case for children with ADHD who do not have NF1 [22–26], attention difficulties have been found to be related to social skills and social problems for children with NF1 [2, 4, 11, 20, 27]. Further, while there is continued debate about the nature of and contributors to ASD symptomatology in NF1 as well as the rate of comorbidity, 13–33% of children with NF1 meet criteria for ASD with frequent report of subclinical symptoms [28–30]. Scores are generally in the mild to moderate range on the Social Responsiveness Scale– Second Edition (SRS-2), a measure commonly used to characterize social responsiveness, social cognition, social awareness and restricted and repetitive behavior related to ASD [27]. Notably, while social difficulties are indeed evident for children with NF1 they are often milder than seen for autistic individuals [28–31].

One prominent program to address the development of social skills and peer relationships in individuals with ASD and other neurodevelopmental conditions associated with social functioning difficulties is *The Program for the Education and Enrichment of Relational Skills* (PEERS®) [32]. PEERS® is a well-validated, caregiver-assisted social skills intervention provided in a group format for children and adolescents who have difficulty making and keeping friends. A number of studies demonstrate the foundational evidence of the PEERS® intervention for autistic children and adolescents using the 14-week in-person intervention [33–35]. Over time, this intervention has been extensively researched and has continued to demonstrate improvements in social knowledge, social responsiveness, social skills, and frequency of get-togethers [36–38]. These domains are particularly relevant as research has shown social competence [39], social responsiveness [27], and social skills [4] are areas of difficulty for individuals with NF1. The promise of this intervention among other populations has also been shown in individuals with ADHD [40] and in adolescents with brain injury [41].

The PEERS® curriculum was recently adapted to be offered virtually through telehealth. Adaptations from the in-person curriculum included recorded role play videos and homework assignment modifications to allow for virtual social opportunities. While the evidence of telehealth PEERS® is still in progress, there has been promising pilot data from several groups to suggest improvement in peer relationships from participation in the intervention delivered in the telehealth modality. The telehealth delivery of PEERS® has shown improvements in social skills knowledge, social responsiveness, social skills, number of adolescent reported get-togethers, and comparable findings to in-person outcomes [42, 43]. Importantly, research on other telehealth interventions within rare populations such as with Prader-Willi syndrome, Williams syndrome, and NF1 over the last five years have pointed to the promise of telehealth approaches with individuals with rare neurogenetic conditions [13, 14, 44, 45].

The lack of research on social skills interventions is likely to be due to rarity of NF1 which presents challenges to conducting face-to-face intervention work with weekly sessions for an extended period of time (e.g., for 14 weeks as in the PEERS® intervention). However, the indication that telehealth interventions may be useful in children and adolescents with other genetic neurodevelopmental conditions [44, 45] and the now available telehealth approach for the PEERS® intervention makes exploration of this intervention within NF1 feasible. The central aim of the proposed research was to conduct a pilot study to provide proof of concept of the effectiveness of a 14-week telehealth-delivered PEERS® intervention to improve social skills and peer interactions for adolescents with NF1. It is hypothesized that adolescents with NF1 who complete the PEERS® intervention (with a parallel caregiver group) will show improvement from pre-test to post-test and maintain improvements at follow-up.

## Materials and methods

### Participants

**Fig. 1:**
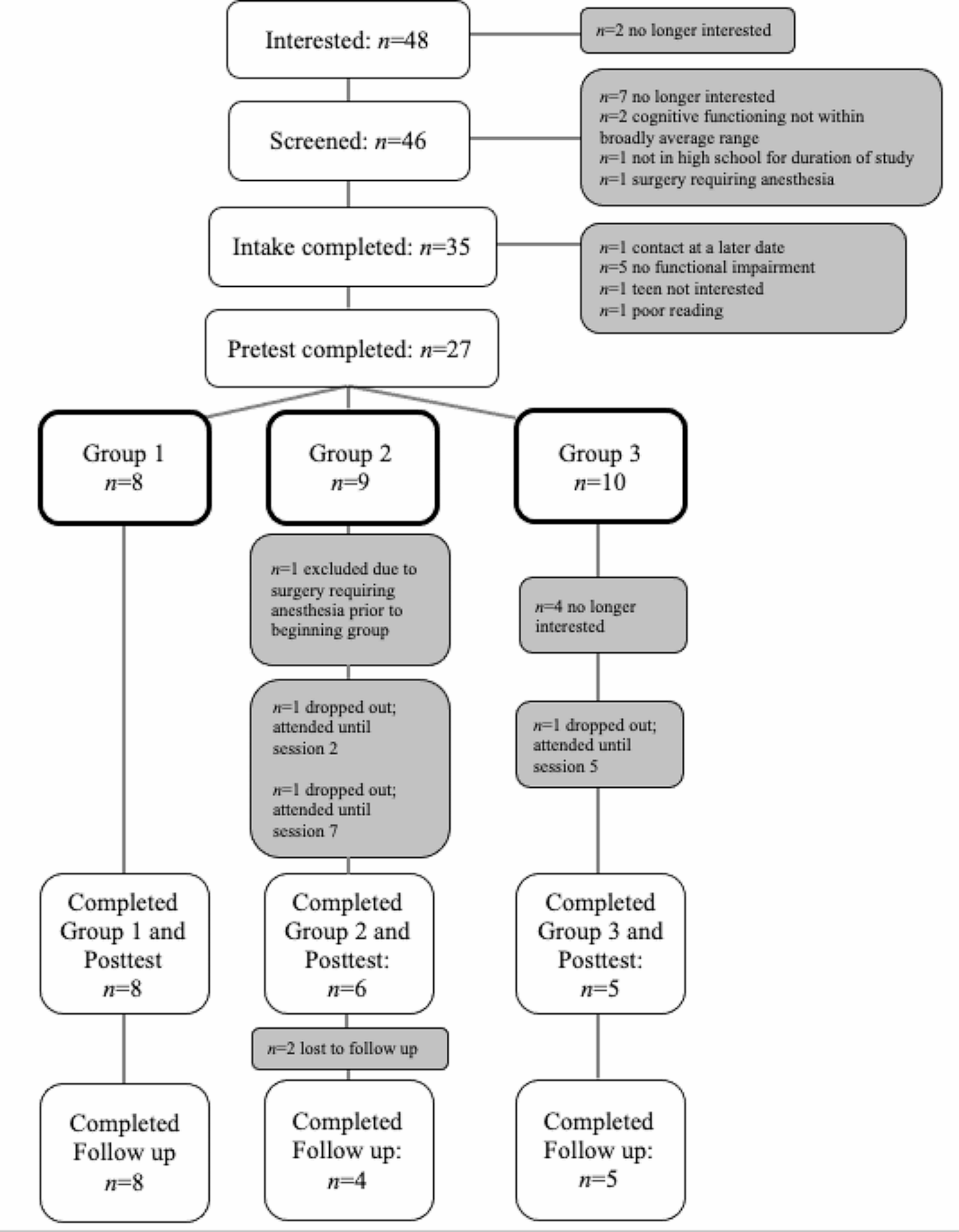
Flow of study participants from recruitment to follow-up. Reason for exclusion indicated when applicable. Participants who dropped out are also specified

Participants were 19 adolescents with NF1 with social skills difficulties and at least one caregiver from each family. Demographic information, sample characteristics, and pre-test (i.e., baseline) outcome data are reported in Table 1. Figure 1 illustrates the flow of participants.

**Table 1:** Demographic information and mean scores on outcome measures for all participants (*n* = 19)

Variable (Mean (SD))											
**Age (at time of entry into the study)**	14.21 (1.63)										
**Participant Reported Sex or Gender**	Male– 12 (63%) Female– 7 (37%)										
**Race/Ethnicity**	White– 15 (79%) Biracial– 2 (10.5%) Asian– 2 (10.5%)										
**NF Etiology**	Familial– 2 (10.5%) Sporadic– 17 (89.5%)										
**KBIT-2 SS**	100.21 (14.07)										
**WIAT-III Word Reading SS**	94.21 (12.41)										
**SCQ Lifetime Raw**	5.95 (4.45)										
**ADHD Diagnosis**	No Diagnosis– 8 (42%) Inattentive Type– 8 (42%) Combined Type– 2 (11%) Diagnosis Missing– 1 (5%)										
				*Pretest– Posttest*				*Pretest– Follow-Up^*			
**Timepoints**	Pretest	Posttest	Follow-Up^	*t*	*df*	*p*	*g*	*t*	*df*	*p*	*g*
**SSIS-SEL-P SS**	84.47 (10.87)	*89.42 (11.61)*	*91.18 (12.84)*	-2.25	18	0.018*	− 0.49	-2.34	16	0.016*	− 0.54
**SSIS-SEL-S SS**	*96.58 (14.79)*	*98.11 (13.68)*	101.29 (13.12)	− 0.69	18	0.25	− 0.15	-1.56	16	0.07*	− 0.36
**SRS-2 T-score**	65.47 (9.29)	60.74 (9.93)	*59.35 (10.61)*	2.85	18	0.005*	0.63	3.33	16	0.002*	0.77
**TASSK Raw**	15.21 (2.74)	21.37 (3.73)	21.18 (4.35)	-8.34	18	< 0.001*	-1.83	-6.54	16	< 0.001*	-1.51
**QSQ-Caregiver Raw**	1.74 (1.88)	3.16 (1.97)	4.12 (3.53)	-3.85	18	< 0.001*	− 0.85	-2.81	16	0.006*	− 0.65
**QSQ-Adolescent Raw**	4.32 (5.77)	5.42 (5.68)	6.65 (8.08)	− 0.97	18	0.17*	− 0.21	-1.16	16	0.13*	− 0.27
**FQS Raw**	84.58 (17.78)	86.63 (12.75)	88.88 (15.59)	− 0.49	18	0.32	− 0.11	-1.22	16	0.12*	− 0.28

Note SD: Standard Deviation; KBIT-2: Kaufman Brief Intelligence Test– Second Edition; SS: Standard Score; WIAT-III: Wechsler Individual Achievement Test– Third Edition; SCQ: Social Communication Questionnaire; ADHD: Attention-Deficit/Hyperactivity Disorder; SSIS-SEL-P: Social Skills Improvement System Social-Emotional Learning Parent Form; SSIS-SEL-S: Social Skills Improvement System Social-Emotional Learning Student Form; SRS-2: Social Responsiveness Scale– Second Edition; TASSK: Test of Adolescent Social Skills Knowledge; QSQ: Quality of Socialization Questionnaire; FQS: Friendship Qualities Scale. Higher standard scores (SS; Mean = 100, SD = 15) and higher raw scores indicate better abilities. Higher T-scores (Mean = 50, SD = 10) indicate more impairment. ^ denotes *n* = 17; *Italics* = Standardized measure with a mean score within 1 standard deviation; **Bold** = *p* <.05.; * denotes *p* <.20 (Lee et al., 2014)

### Procedure

This study was approved by the University of Wisconsin-Milwaukee’s Institutional Review Board. Recruitment methods included (1) contacting previous NF1 participants who have agreed to be contacted about future research studies, (2) providing fliers to surrounding area Neurofibromatosis clinics, and (3) announcements in the Children’s Tumor Foundation patient NF Registry system and NF foundation newsletters (e.g., NF Midwest, NF Northeast). Inclusion and exclusion criteria are included in Table 2 with certain criteria suggested by the PEERS® curriculum [32]. All study activities took place virtually via phone and/or online video conferencing platforms (e.g., Zoom or Microsoft Teams). Assessments and questionnaire measures were administered by well-trained graduate students or other professionals. Interested participants were screened for eligibility criteria over the phone. Following screening, they were consented/assented for a caregiver and adolescent intake session to further determine eligibility and describe the intervention details. The caregiver portion of the intake session involved obtaining the caregiver’s perspective of their adolescent’s social participation. The adolescent portion of the intake session included obtaining the adolescent’s perspective on their social participation, exploring their interest and motivation in the intervention, and brief assessments of cognitive functioning and reading ability. Once eligibility was determined, participants were consented/assented to participate in the study. A pre-test session was conducted with both the caregiver and the adolescent that included administration of electronic questionnaires focused on the outcomes of interest. Caregivers also completed a semi-structured interview of ADHD symptomatology with a trained study staff member.

**Table 2:** Inclusion and exclusion criteria for study participants

Inclusion Criteria	Exclusion Criteria
Diagnosis of neurofibromatosis type 1 (NF1) by a physician	Any cognitive or developmental delays that would affect reading comprehension and/or understanding of the treatment material by caregiver report and assessed using the Kaufman Brief Intelligence Test– Second Edition and Wechsler Individual Achievement Test– Third Edition Word Reading subtest during intake
Ages 12–17	First language and main language spoken in the home is not English (because standardized study measures and instructions are all in English)
In middle school or high school for duration of the study	Any significant behavioral concerns (e.g., violence, need of a one-on-one aide)
First language and main language spoken in the home is English	Any other comorbid medical conditions not commonly associated with NF1 (e.g., other illnesses; hearing or vision impaired)
Reliable access to internet	A major surgery (e.g., brain or heart surgery) within the past six months
Exhibit current functional impairment in peer relationships, which will be confirmed during the caregiver interview	Adolescent does not agree to participate or attend voluntarily
Willingness to attend all treatment sessions, with a maximum of two allowed absences	Prior social skills group treatment within the past six months
Interested in and motivated to participate in the treatment, evaluated during a structured interview at pre-assessment	

The PEERS® intervention was administered by certified PEERS® providers when possible or by well-trained graduate students or other professionals according to the PEERS® teleconference training provided by Dr. Elizabeth Laugeson of the University of California-Los Angeles in conjunction with the PEERS® manual [32] and the CARD telehealth manual (*PEERS Remote Manual*, n.d.). Supervision was provided by a Licensed Psychologist who is a certified PEERS® provider. The PEERS® intervention involves separate caregiver and adolescent sessions that meet for 90 min each week for a 14-week period. For a summary of PEERS® sessions, see Laugeson and colleagues [34].

Within two weeks of the final session of the intervention (which consisted primarily of a graduation ceremony), adolescents and caregivers again completed a set of electronic questionnaires (post-test). Mean time between pre-test and post-test was 111.47 days (15.92 weeks); SD = 5.94 days. A 14-week (± 2 weeks) follow-up was also conducted where a set of electronic questionnaires was administered again (for a third time). Mean time between pre-test and follow-up was 221.27 days (31.61 weeks); SD = 33.93 days (4.85 weeks) and mean time between post-test and follow-up was 94.83 days (13.54 weeks); SD = 10.57 days (1.51 weeks). Families received compensation for completing and submitting electronic questionnaires at follow-up.

### Measures to describe sample characteristics

*Kaufman Brief Intelligence Test– Second Edition* (*KBIT-2)* [46]. The KBIT-2 is a brief measure of cognitive abilities including verbal reasoning and nonverbal reasoning. The KBIT-2 demonstrates good reliability and validity. Telehealth administration guidelines of the KBIT-2 were followed. Verbal reasoning is assessed based on two subtests (receptive vocabulary and word-reasoning using “riddles”). Nonverbal reasoning is assessed using a matrices task (i.e., determining which picture completes a puzzle). The IQ composite, verbal and nonverbal standard scores (*M* = 100, *SD* = 15) were used to characterize cognitive functions and aid in eligibility determination for the study. Higher scores represent higher cognitive abilities. Cognitive abilities in the broadly average range or above were considered meeting eligibility criteria. This measure was administered at the adolescent intake appointment.

*Wechsler Individual Achievement Test– Third Edition (WIAT-III) Word Reading subtest* [47]. The WIAT-III is a measure used to assess academic functioning throughout childhood and adolescence. The WIAT-III Word Reading subtest examines the ability to accurately read words. This subtest standard score (*M* = 100, *SD* = 15) was used to characterize reading abilities and aid in eligibility determination for the study. Higher scores represent better reading abilities. Reading abilities in the broadly average range or above were considered meeting eligibility criteria. This measure was administered at the adolescent intake appointment.

*Schedule for Affective Disorders and Schizophrenia for School-Age Children-Present and Lifetime Version– Attention Deficit/Hyperactivity Disorder module (KSADS-ADHD)* [48]. The KSADS is a semi-structured caregiver interview to assess psychopathology in children and adolescents according to the Diagnostic and Statistical Manual-Fifth Edition criteria. Strong reliability and validity have been demonstrated [48]. The KSADS-ADHD module includes assessment of inattention, hyperactivity and impulsivity symptoms and was administered to determine if participants met research criteria for an ADHD diagnosis. Symptom descriptions are rated by the interviewer on a 4-point scale including “No information,” “Not present,” “Occurs occasionally,” and “Occurs often.” This interview was administered at the pre-test appointment to a caregiver.

*Social Communication Questionnaire (SCQ)* [49]. The SCQ is a caregiver-report questionnaire that examines ASD symptomatology. The SCQ demonstrates good reliability and validity [50, 51]. The SCQ has 40 items that are rated as “Yes” or “No.” The SCQ yields a raw score that is compared to specific research cutoff score (e.g., cutoff of 15 indicates the possibility of ASD). Higher scores represent more ASD symptomatology. This questionnaire was administered at pre-test to a caregiver.

### Outcome measures

#### Social functioning

*Social Skills Improvement System Social-Emotional Learning* (*SSIS-SEL)* [52]. The SSIS-SEL is a caregiver and self-report questionnaire measure examining social-emotional skills in childhood and adolescence administered at each of the three timepoints (pre-test, post-test, and follow-up). The SSIS-SEL is the most recent update to the Social Skills Rating System (SSRS) and Social Skills Improvement System (SSIS). Prior versions of this measure have been widely used within the NF1 literature [4–7, 10, 53, 54] and have been recommended as an outcome for social functioning in NF1 [55]. While the number of subscales and the subscale names on the SSIS-SEL have evolved from prior versions of this measure, the social skills items on this measure have remained consistent and the social skills composite scores are highly correlated (*r* =.97) [52, 56]. Adequate internal consistency, test-retest reliability, and validity have been demonstrated by the measure developers [53, 57]. The Parent (SSIS-SEL-P; 51 items) and Student (SSIS-SEL-S; 46 items) forms were used for the caregiver and adolescent, respectively. The reporter is asked to rate each item using a 4-point scale including “Never,” “Seldom,” “Often” and “Almost Always.” Higher scores represent better social-emotional skills. The Social-Emotional Learning (SEL) composite standard score (*M* = 100, *SD* = 15) was used to assess social-emotional skills and as a measure of the effectiveness of the intervention. In the current sample, internal consistency for the SEL composite standard score was good and excellent, respectively, for both the Parent and Student forms (Parent: α = 0.86; Student: α = 0.96). Standard scores of SSIS-SEL subscales are also available and were examined on the Parent and Student forms with internal consistencies in the current sample indicated in parentheses (Self-Awareness (SeA; Parent: 7 items, α = 0.67; Student: 9 items, α = 0.81), Self-Management (SM; Parent: 14 items, α = 0.64; Student: 9 items, α = 0.75), Social Awareness (SA; Parent: 7 items, α = 0.89; Student: 7 items, α = 0.85), Relationship Skills (RS; Parent: 14 items, α = 0.61; Student: 15 items, α = 0.91), and Responsible Decision Making (RDM; Parent: 9 items, α = 0.81; Student: 6 items, α = 0.66)). For analyses examining frequency of social difficulties on this measure, difficulty is represented by a standard score below 85, which is consistent with the SSIS-SEL manual cut-off for below average skills that may require intervention.

*Social Responsiveness Scale– Second Edition* (*SRS-2)* [57]. The SRS-2 is a caregiver-report questionnaire of social impairment and repetitive behavior (ASD symptomatology) in childhood and adolescence administered at each of the three timepoints (pre-test, post-test, and follow-up). Adequate internal consistency, test-retest reliability, and validity have been demonstrated by the developers. The measure includes 65 items that are rated on a 4-point scale including “Not true,” “Sometimes true,” “Often true,” and “Almost always true.” Higher scores represent more social responsiveness challenges and more social impairment. Given the Total Score is the most well-researched score from the SRS-2, the Total Score T-score (*M* = 50, *SD* = 10) was examined and used as a measure of the effectiveness of the intervention. In the current sample, internal consistency for the SRS-2 Total Score T-score was excellent (α = 0.93). Treatment social subscales are available and were also examined with internal consistencies within this sample indicated in parentheses (Social Awareness (Saw; 8 items, α = 0.72), Social Cognitive (Scog; 12 items, α = 0.79), Social Communication (Scom; 21 items, α = 0.47), and Social Motivation (Smot; 11 items, α = 0.82) as well as the DSM-5 compatible scale of Social Communication and Interaction (SCI; 53 items, α = 0.91). Importantly, the measure developers suggest that the application of treatment subscales should be limited to investigations specific to alleviation of symptoms such as when evaluating treatment effects [57]. Internal consistencies were also calculated within this sample and are indicated in parentheses. Difficulty was represented by a T-score above 60, which is consistent with the SRS-2 manual cut-off for mild deficits.

*Friendship Qualities Scale* (*FQS)* [58]. The FQS is a self-report questionnaire examining the friendship qualities of companionship, conflict, help, security and closeness within a friendship and was administered at each of the three timepoints (pre-test, post-test, and follow-up). During development of this questionnaire, Bukowski and colleagues reported adequate internal consistency and validity. The adolescent is asked to think of their friendship with their closest friend and rate 23 items using a 5-point scale including “Not true,” “A little true,” “Somewhat true,” “Mostly true” and “Really true.” The FQS yields a total score ranging from 0 to 115 with higher total scores indicating better quality friendships. In the current sample, internal consistency for the FQS total score was high (α =.92). The FQS total score was used to gather information about the adolescent’s quality of friendships.

*Quality of Socialization Questionnaire* (*QSQ)* [59]. The QSQ is a caregiver and self-report questionnaire to gather information about the adolescent’s get-togethers with peers at each of the three timepoints (pre-test, post-test, and follow-up). Caregivers and adolescents are asked about how many get-togethers the adolescent organized (1 item; i.e., social initiation) and how many get-togethers the adolescent was invited to in the last month (1 item; i.e., social reciprocity); there are also questions about conflict during the get-togethers (12 items), which were not analyzed. Adequate discriminant validity between community and clinic samples and high inter-rater (parent and teen) correlations have been demonstrated in a sample of teenagers with ASD [33]. Combined raw scores on the social initiation and social reciprocity items (i.e., number of total get togethers) were examined with higher raw scores representing more get-togethers.

*Test of Adolescent Social Skills Knowledge* (*TASSK)* [33]. The TASSK is a self-report 30-item questionnaire that measures knowledge of specific social skills taught as part of the PEERS® intervention and is administered to adolescents at each of the three timepoints (pre-test, post-test, and follow-up). Adequate internal consistency (α = 0.56) has been reported in a sample of teenagers with ASD [35]. Higher scores represent greater knowledge of the PEERS® social skills curriculum.

### Statistical analysis

The data were analyzed using IBM SPSS for Windows, version 28. A post-hoc power analysis was conducted using GPower 3.1 (*n* = 19) [60]. Under a typical one-tailed 0.05 criterion of statistical significance, the study is underpowered to detect differences for small and medium effect sizes (0.21 and 0.67) and adequately powered for large effect sizes (0.96). However, the pilot nature of this study suggests an alternative statistical threshold, such as 0.20 criterion for statistical significance, may be adopted to demonstrate initial efficacy [61]. Thus, findings within the tables that demonstrate initial efficacy using *p* <.20 criterion for statistical significance are highlighted for additional context, though we interpret the more conservative (*p* <.05) significant findings within the results and discussion. With a one-tailed 0.20 criterion of statistical significance, the study is underpowered to detect differences for small effect sizes (0.51) and adequately powered for medium and large effect sizes (0.91 and 0.99).

One sample t-tests examined ratings on norm-referenced measures in comparison to the normative mean. Paired samples t-tests compared performance across the three timepoints (pre-test, post-test, and follow-up). Findings were interpreted with respect to both statistical significance and effect size. Effect size (Hedges g’) interpretations are as follows: negligible effect = 0– 0.14; small effect = 0.15– 0.39; medium effect = 0.40– 0.74; large effect ≥ 0.75.

Initially, data were analyzed using both a completer analytic approach (i.e., data only from participants who completed the intervention) and an intent to treat approach with the last observation carried forward method to account for the missing data for individuals who drop out of a study before completion. Specifically, if an individual dropped out during the current intervention, the pre-test score was used as their post-test score. However, completer analyses are often used even when drop out is present in the PEERS® literature [33, 40, 62]. Thus, to match that of the existing PEERS® literature, results of the completer analyses are presented here. Notably, the results were generally similar for all group-level analyses across the completer and intent-to-treat approaches.

## Results

Adolescents with NF1 had significantly lower overall social-emotional skills and significantly more social impairment using caregiver report compared to the normative mean with large effect sizes at pre-test (SSIS-SEL-P: *t*(18) = -6.23, *p* <.001, *g* = -1.37; SRS-2: *t*(18) = 7.26, *p* <.001, *g* = 1.59). Self-report of overall social-emotional skills was not significantly different from the normative mean at pre-test. With standard scores of < 85 classified as a difficulty and ≥ 85 as no difficulty, difficulties were reported for seven participants on the SSIS-SEL-P and four participants on the SSIS-SEL-S at pre-test. Fourteen participants were reported to have difficulty on the caregiver-reported SRS-2 at pre-test with a T-score of > 60 defined as difficulty.

Caregiver-reported social-emotional skills (SSIS-SEL-P) and social impairment (SRS-2) were significantly better at post-test and follow-up compared to pre-test with medium effect sizes (Table 1). Caregiver-reported number of adolescent get-togethers (QSQ-Caregiver) and social knowledge (TASSK) were also significantly better at post-test and follow-up compared to pre-test with medium to large effect sizes. Self-reported social-emotional skills (SSIS-SEL-S), quality of existing friendships (FQS), and adolescent-reported number of get-togethers (QSQ-Adolescent) were not significantly different from pre-test to post-test or follow-up.

Mean ratings on the SSIS-SEL Parent Form subscales were significantly lower compared to the normative mean across all subscales with medium to large effect sizes (Table 3). On the SSIS-SEL Parent Form, ratings on the subscales of Self-Management and Relational Skills were significantly improved at post-test with medium effect sizes. Caregiver ratings on the SRS-2 subscales were significantly elevated compared to normative data with large effect sizes. All subscales on the SRS-2 were significantly better at post-test with medium effect sizes (Table 3). The Relational Skills subscale on the SSIS-SEL Student Form was significantly lower compared to the normative mean with a small effect size. All other subscales on the SSIS-SEL Student form were not significantly different from normative data. The Self-Management subscale on the SSIS-SEL Student Form was significantly better at post-test with a medium effect size while all other SSIS-SEL Student subscales were not significantly different from pre- to post-test.

**Table 3:** Exploration of primary outcome measure subscales (*n* = 19)

Mean (SD)	Pre-test	Post-Test	Follow-Up^	One Sample t-test				Pretest - Posttest				Pretest– Follow-Up^			
***SSIS-SEL-P SS***	Mean	Mean	Mean	*t*	*df*	*p*	*g*	*t*	*df*	*p*	*g*	*t*	*df*	*p*	*g*
Self-Awareness	77.21 (15.55)	81.0 (10.58)	79.18 (11.75)	-6.39	18	**< 0.001**	-1.40	-1.071	18	0.15*	− 0.24	− 0.79	16	0.22	− 0.18
Self-Management	*89.53 (10.29)*	*95.32 (9.03)*	*97.29 (13.01)*	-4.44	18	**< 0.001**	− 0.98	-2.711	18	0.007*	− 0.60	-1.71	16	0.009*	− 0.61
Social Awareness	*91.0 (16.95)*	*93.68 (14.87)*	*98.76 (12.70)*	-2.31	18	**0.016**	− 0.51	− 0.922	18	0.18*	− 0.20	-2.09	16	0.026*	− 0.48
Relationship Skills	*86.89 (11.01)*	*92.95 (12.66)*	*93.71 (14.40)*	-5.19	18	**< 0.001**	-1.14	-2.943	18	0.004*	− 0.65	-2.24	16	0.02*	− 0.52
Responsible Decision Making	*91.32 (16.6)*	*93.37 (14.03)*	*94.53 (14.15)*	-2.28	18	**0.018**	− 0.50	− 0.900	18	0.19*	− 0.19	-1.32	16	0.10*	− 0.30
***SRS-2 T-scores***															
Social Awareness	62.37 (13.68)	*58.42 (10.41)*	*58.18 (9.25)*	3.94	18	**< 0.001**	0.87	1.902	18	0.037*	0.42	1.79	16	0.046*	0.41
Social Cognitive	63.37 (10.35)	*58.74 (11.02)*	*56.88 (10.78)*	5.63	18	**< 0.001**	1.24	2.238	18	0.019*	0.49	2.97	16	0.005*	0.69
Social Communication	65.84 (8.36)	60.68 (9.23)	*59.82 (10.94)*	8.26	18	**< 0.001**	1.81	2.850	18	0.005*	0.63	2.74	16	0.007*	0.63
Social Motivation	60.73 (12.23)	*57.47 (10.69)*	*56.65 (11.92)*	3.86	18	**< 0.001**	0.84	1.914	18	0.036*	0.42	2.28	16	0.018*	0.53
Social Communication and Interaction	65.26 (9.26)	60.37 (9.65)	*59.12 (10.98)*	7.18	18	**< 0.001**	1.58	2.846	18	0.005*	0.63	3.05	16	0.004*	0.70
***SSIS-SEL-S SS***															
Self-Awareness	*99.32 (14.07)*	*100.95 (12.39)*	*101.35 (12.13)*	− 0.21	18	0.42	− 0.05	− 0.691	18	0.25	− 0.15	− 0.93	16	0.18*	− 0.22
Self-Management	*95.79 (13.37)*	*99.89 (14.11)*	*102.06 (13.87)*	-1.37	18	0.09	− 0.30	-1.927	18	0.035*	− 0.42	-2.26	16	0.02*	− 0.52
Social Awareness	*97.63 (14.99)*	*97.37 (15.57)*	*101.65 (14.17)*	− 0.69	18	0.25	− 0.15	0.081	18	0.47	0.018	− 0.97	16	0.17*	− 0.23
Relationship Skills	*92.89 (17.61)*	*93.16 (17.04)*	*96.65 (16.05)*	-1.76	18	**0.048**	− 0.39	− 0.110	18	0.46	− 0.02	-1.11	16	0.14*	− 0.26
Responsible Decision Making	*99.68 (11.93)*	*101.16 (12.5)*	*104.18 (12.03)*	− 0.12	18	0.46	− 0.03	− 0.605	18	0.28	− 0.13	-1.54	16	.07*	− 0.36

Note SD: Standard Deviation; SSIS-SEL-P: Social Skills Improvement System Social-Emotional Learning Parent Form; SS: Standard Score: SSIS-SEL-S: Social Skills Improvement System Social-Emotional Learning Student Form; SRS-2: Social Responsiveness Scale– Second Edition; Higher standard scores (Mean = 100, SD = 15) indicate better abilities. Higher T-scores (Mean = 50, SD = 10) indicate more impairment. ^ denotes *n* = 17; *Italics* = Standardized measure with a mean score within 1 standard deviation; **Bold** = *p* <.05; * denotes *p* <.20 (Lee et al., 2014)

## Discussion

This investigation is the first to examine a specific social skills intervention, PEERS®, for use in adolescents with NF1 with social challenges. This research both utilized an intervention that had not previously been used in NF1 and also explored the use of telehealth delivery among a population with minimal telehealth focused research. Group-level improvements in caregiver-reported social-emotional skills, social impairment, caregiver-reported number of adolescents get-togethers, and adolescent’s social knowledge at post-test and at follow-up were found, consistent with the broad aim of this research. These findings provide initial evidence that the PEERS® intervention is likely to be effective and helpful within this population and is a highly promising resource for medical providers, school personnel, researchers, and clinicians who work with individuals with NF1.

### Improvements in social functioning were observed even in the context of mild social difficulties

Group-level improvements in social functioning on norm-referenced measures were observed following the intervention based on caregiver report, even though the majority of individuals with NF1 (63%) in this sample showed broadly average range social challenges at baseline. It is notable that these mild social difficulties were reported on standardized measures of social function at pretest even though the PEERS® intake and eligibility process include that families indicate a desire to improve their adolescent’s social relationships, concern regarding their social functioning, and evidence that they do not have many close friendships. There were indeed several participants who were excluded due to social challenges that were not substantial enough for inclusion in the group based on caregiver and/or adolescent report. Generally mild social difficulties, with means similar to those seen here, have indeed been described in the literature previously [4, 53, 54] and yet the overall scores on the SSIS-SEL and SRS-2 are likely to adequately capture change with intervention, as observed in this pilot study.

To further understand how to best evaluate the impact of PEERS®, the current investigation also explored whether certain subscales on the standardized outcome measures would be more apt to capture change from the intervention. PEERS® research with autistic adolescents using previous versions of the SSIS-SEL (i.e., Social Skills Rating System (SSRS) and Social Skills Improvement System (SSIS)) and SRS-2 has shown inconsistent findings at the subscale level, with one reporting no improvements at the subscale level [37] and another reporting significant subscale improvements [34]. The current study found SSIS-SEL-P and SRS-2 caregiver subscale ratings and SSIS-SEL-S Relational Skills subscale indicated significant difficulties in comparison to the normative mean at baseline. SSIS-SEL-P Self-Management and Relational Skills subscale, and all SRS-2 subscales showed improvement following the intervention. These findings indicate that continued inclusion and exploration at the subscale level is warranted.

### Self-report of social functioning was less likely to capture improvements

Self-report was utilized in the current study to understand how the adolescent may view their skills and capture their experience. Caregiver’s perception of skills may be different than the adolescent’s social experience themselves as is evident in the literature on bullying victimization in NF1 [63]. Self-report of social skills or social-emotional skills is not utilized in the majority of PEERS® research [33–35, 37, 38]. Self-report measures (e.g., examination of get-togethers with QSQ, and quality of existing friendships with FQS) have shown variability in their change following the PEERS® intervention with some studies finding improvement based on teen self-reported outcomes [33–35, 37, 38, 40, 41, 62] and others not [38, 40]. In the current study, it was rare for the teen self-report ratings to indicate difficulties in comparison to same-aged peers, which may serve as a protective factor against feelings of inadequacy and negative interactions with others [64, 65]. Similarly, adolescents with NF1 have been found to overestimate abilities (e.g., academic achievement) [4, 66]. However, the teens did show increased knowledge of social skills concepts that are directly taught within this curriculum and improvements on the SSIS-SEL-S Self-Management scale. The Self-Management SSIS-SEL-S subscale largely assesses managing emotions (e.g., staying calm during disagreements) and social stress (e.g., teasing) [52], which are both direct targets of the PEERS® intervention. Self-reported quality of existing friendships and number of get-togethers demonstrated change in the positive direction following the intervention at post-test and follow-up, though with negligible and small effect sizes respectively. Using a less stringent criterion (*p* <.20) that can be appropriate for pilot studies of initial efficacy [61], most adolescent self-report measures would be significantly improved following the intervention at follow-up.

### Telehealth is a promising direction for intervention in NF1

Telehealth interventions may hold particular promise among rare conditions such as NF1 where a face-to-face intervention would not be feasible. Telehealth delivered services are generally well-received by patients [67], with broad benefits in increased access to services, convenience and flexibility of this modality, cost-effectiveness, and higher quality care and life [68–70] as well as documented benefits specifically in NF1 [71]. Critiques of telehealth from provider’s perspectives, in general, have included concerns for maintaining relationships, security breaches and confidentiality, audio and video difficulties, legal issues, reimbursements and administrative burden [72, 73]. However, health care workers have described an overall positive impression in utilizing telehealth to provide NF related care [74]. These results support the budding telehealth research in the NF1 population, particularly with adolescents with NF1 and their families.

### Considerations, limitations, and future directions

There are several considerations and limitations of this pilot research. First, this intervention is group-based with caregiver involvement rather than individualized to fit each participant’s specific needs. Second, this intervention is based on neurotypical assumptions and may not promote the ongoing shift towards neurodiversity [75]. Third, fidelity checks of the intervention were not performed at this pilot stage in the research. Fourth, while the sample size of the current pilot study is similar to other studies using PEERS® [33, 34, 40, 62], it is still small, resulting in underpowered analyses. There are very few participants with a familial NF etiology which is not representative of the NF population as a whole. This sample is primarily White and there are no Latinx participants which limits the generalizability of these findings to additional racial and ethnic backgrounds. There is a need for attention in the field to methods of participant engagement that increases the diversity and representativeness of clinical research samples. Nevertheless, this is the first investigation, to our knowledge, to explore a telehealth social skills intervention in NF1 and one of very few exploring the telehealth format of the PEERS® intervention.

This research endeavor would not have been possible without the increase in use and acceptance of telehealth over the last several years which presented a unique opportunity to explore a virtual social skills intervention among this population. While telehealth is widely used, it is important to consider the context in which this investigation took place (during the COVID-19 pandemic) while keeping in mind the novelty of this approach. The pandemic context could have resulted in differential impact for individual participants as well as across the study as a whole. Specifically, the fluctuating total and new cases of COVID-19 per day, varying federal and state requirements, and dissemination of a COVID-19 vaccination likely resulted in changes to how often participants engaged in social interactions (i.e., some families may have participated more in social distancing while others participated in-person activities). Additionally, it is likely that several families experienced common stressors of the COVID-19 pandemic such as changes in caregiver employment or finances as well as exposures to and contracting COVID-19 during the group. These differential experiences would be most likely to result in variable responses on the QSQ, or the measure of get-togethers, within this investigation, although additional exploration is needed. However, the relatively low drop out from the intervention experienced amidst the COVID-19 pandemic are rather remarkable. Lastly, extracurricular activities, get-togethers, and homework assignments were allowed to occur virtually given the context and methodology of the current study. This research team assisted with identifying potential local or national online extracurricular options for families who reported difficulty. Similarly, with virtual get-togethers, families were often provided with suggestions for videoconferencing platforms as well as online games or activities that could be conducted virtually. While this level of problem-solving may not be present in the in-person PEERS® protocol, it appeared crucial to the telehealth-delivered PEERS® for participants to be able to participate in activities that most emulate the in-person version of this intervention. Finally, this telehealth-delivered intervention also provided an opportunity for families with NF1 to interact and experience this intervention together. The rarity of NF1 makes it unlikely for families to know other families with NF1 and thus, it may be the case that this intervention provides a unique opportunity for families with NF1 to meet each other when they otherwise would not have, but additional exploration of this notion is required.

This pilot research has also pointed to several future directions. Broadening with a larger sample size is necessary to appropriately capture change from this intervention (e.g., provide more power to detect differences in self-report measures; examine individual outcomes rather than group-level outcomes), assess contributors to differential outcomes (e.g., NF1 physical features, cognitive functioning, ADHD status or level of social skills challenges on norm-referenced assessments), and control for drop out and missing data using sophisticated statistical techniques. Expansion and replication of this study design will also help to determine how to best capture adolescent’s experience with social skills and identify more sensitive or targeted measures to highlight their social experiences (e.g., rating adolescent behavior and skills using a behavior rating system during the group). Future directions also include inquiring more specifically about the impact of the intervention on bullying experiences, as there is indication that individuals with milder social difficulties experience more bullying and victimization compared to more socially impaired individuals [76]. A multisite collaboration would be a beneficial method next step for several reasons: to demonstrate feasibility of other research teams conducting the study procedures; to aid recruitment and participation efforts for a sufficiently powered randomized controlled trial; allow researcher blinding during outcome measure administration; and to serve as a comparison group to the current sample.

This research points to the promise of the telehealth PEERS® intervention strategy for improving social and friendship skills among adolescents with NF1 who have social difficulties. The telehealth modality may be particularly useful given the rarity of NF1 and the limited number of teens with NF1 expected to be within driving distance of an in-person provider familiar with NF1. Additionally, given our findings of positive impact of this curriculum, families who desire an in-person option may also seek in-person PEERS® interventions near them, together with teens with other neurodevelopmental conditions associated with social difficulties, given the widespread availability of this intervention.

## Data Availability

The datasets generated and analyzed during the current study will soon be publicly available through Synapse.

## Abbreviations

NF1: neurofibromatosis type 1
ADHD: attention-deficit/hyperactivity disorder
ASD: autism spectrum disorder
SSRS: Social Skills Rating System
SSIS: Social Skills Improvement System
SRS-2: Social Responsiveness Scale– Second Edition
PEERS®: The Program for the Education and Enrichment of Relational Skills
KBIT-2: Kaufman Brief Intelligence Test– Second Edition
WIAT-III: Wechsler Individual Achievement Test– Third Edition
KSADS-ADHD: Schedule for Affective Disorders and Schizophrenia for School-Age Children-Present and Lifetime Version– Attention Deficit/Hyperactivity Disorder module
SCQ: Social Communication Questionnaire
SSIS-SEL: Social Skills Improvement System Social-Emotional Learning
FQS: Friendship Qualities Scale
QSQ: Quality of Socialization Questionnaire
TASSK: Test of Adolescent Social Skills Knowledge
